# A Strong Synergy Between the Thiopeptide Bacteriocin Micrococcin P1 and Rifampicin Against MRSA in a Murine Skin Infection Model

**DOI:** 10.3389/fimmu.2021.676534

**Published:** 2021-07-02

**Authors:** Kirill V. Ovchinnikov, Christian Kranjec, Amar Telke, Morten Kjos, Tage Thorstensen, Siegfried Scherer, Harald Carlsen, Dzung B. Diep

**Affiliations:** ^1^ Faculty of Chemistry, Biotechnology and Food Science, Norwegian University of Life Sciences, Ås, Norway; ^2^ ARD Innovation AS, Ås, Norway; ^3^ TUM School of Life Sciences, Technical University of Munich, Munich, Germany

**Keywords:** MRSA, bacteriocin, skin infection bacteria, micrococcin P1, murine model, Rifampi(ci)n

## Abstract

Antibiotic-resistant bacterial pathogens have become a serious threat worldwide. One of these pathogens is methicillin-resistant *Staphylococcus aureus* (MRSA), a major cause of skin and soft tissue infections. In this study we identified a strain of *Staphylococcus equorum* producing a substance with high antimicrobial activity against many Gram-positive bacteria, including MRSA. By mass spectrometry and whole genome sequencing the antimicrobial substance was identified as the thiopeptide bacteriocin micrococcin P1 (MP1). Based on its properties we developed a one-step purification protocol resulting in high yield (15 mg/L) and high purity (98%) of MP1. For shorter incubation times (5-7 h) MP1 was very potent against MRSA but the inhibitory effect was overshadowed by resistance development during longer incubation time (24h or more). To overcome this problem a synergy study was performed with a number of commercially available antibiotics. Among the antibiotics tested, the combination of MP1 and rifampicin gave the best synergistic effect, with MIC values 25 and 60 times lower than for the individual drugs, respectively. To assess the therapeutic potential of the MP1-rifampicin combination, we used a murine skin infection model based on the use of the multidrug-resistant luciferase-tagged MRSA strain Xen31. As expected, neither of the single antimicrobials (MP1 or rifampicin) could eradicate Xen31 from the wounds. By contrary, the MP1-rifampicin combination was efficient not only to eradicate but also to prevent the recurrence of Xen31 infection. Furthermore, compared to fucidin cream, which is commonly used in skin infection treatments, MP1-rifampicin combination was superior in terms of preventing resistance development. Our results show that combining MP1, and probably other thiopeptides, with antibiotics can be a promising strategy to treat SSTIs caused by MRSA and likely many other Gram-positive bacteria.

## Introduction

Skin and soft tissue infections (SSTIs) are among the most common infections in the world and the majority of them is caused by *Staphylococcus aureus* – a major bacterial human pathogen known for its antibiotic resistance and virulence (1, 2). Methicillin-resistant *S. aureus* (MRSA) is of particular concern, since patients with SSTIs caused by MRSA have higher risk of bacteremia, hospital re-admission and death, and often require longer and more expensive periods of hospitalization compared to patients infected with non-MRSA (3, 4). European guidelines recommend vancomycin, teicoplanin, linezolid, daptomycin, tigecycline or ceftaroline for the treatment of MRSA infections (5), however resistance development to these antibiotics has already been reported (6–11). Consequently, there is an urgent need for novel antimicrobial agents and strategies to overcome MRSA in SSTIs.

Thiopeptides are sulfur-containing, ribosomally-produced and highly posttranslationally modified bacteriocins – antimicrobial peptides produced by bacteria to inhibit other bacteria in competition for nutrients and habitats (12, 13). These peptides represent a promising class of natural antibacterial molecules, being active against many Gram-positive pathogens, including antibiotic resistant derivatives such as MRSA, vancomycin-resistant enterococci (VRE) and penicillin-resistant *Streptococcus pneumoniae* (14, 15). Thiopeptides inhibit protein synthesis in sensitive bacteria by binding to a cleft between the ribosomal protein L11 and the 23S rRNA, known as the GTPase-associated center, or by binding to and inactivating the elongation factor Tu (16–18). Besides their antimicrobial properties, some thiopeptides have demonstrated antiplasmodial, antifungal and anticancer activities (19–21). In contrast to non-ribosomally synthesized peptides, thiopeptides are encoded by classical structural genes and synthesized ribosomally, which renders the generation of new analogs by genetic engineering relatively straight-forward (22). These facts, combined with low cytotoxicity of thiopeptides (23) make this class of molecules very appealing for clinical use.

More than one hundred thiopeptides have been discovered so far with most of these molecules being produced by soil bacteria, including *Bacillus* spp, *Streptomyces* spp. and *Nocardiopsis* spp (22). However, despite the great therapeutic potential, their low aqueous solubility and the fact that sensitive bacteria can easily develop resistance to these antimicrobials are major drawbacks that have hindered their introduction to clinical practice (16, 18).

Micrococcin P1 (MP1), which was the first discovered thiopeptide, is a hydrophobic and heat-stable molecule with high activity against a wide range of Gram-positive bacteria as well as *Mycobacterium tuberculosis* (24). Interestingly MP1 has been shown to be produced by bacteria from different genera, including *Micrococcus*, *Staphylococcus* and *Bacillus* spp. These bacteria are mostly isolated from soil (22), but also from other sources, e.g., French Raclette cheese (25).

In this study we describe a new producer of MP1 with a novel gene cluster. To increase its antimicrobial activity and to circumvent the problem of bacterial resistance development, we explored the synergy of MP1 with several antibiotics and found that it had indeed a strong synergy with some commonly used antibiotics *in vitro*. Furthermore, we validated this synergistic effect in a murine model of MRSA skin infection.

## Materials and Methods

### Bacterial Strains and Growth Conditions

All bacterial strains used in this study are listed in **Table 1** and **Supplemental Table 1**. *S. equorum* KAVA and *S. equorum* WS 2733 were MP1 producers; the former obtained from this study while the latter from a previous study (25). For *in vivo* imaging of bacterial infection in mice and antimicrobial synergy study, *S. aureus* Xen31 (Perkin Elmer, Waltham, MA) was used. The strain was derived from the parental strain *S. aureus* ATCC 33591, a clinical MRSA isolated from Elmhurst Hospital in New York (26). *S. aureus* Xen31 possesses a stable copy of the modified *Photorhabdus luminescens luxABCDE* operon at a single integration site on the bacterial chromosome. To define the inhibition spectrum of MP1, a panel of bacteria from different genera and species were used (see **Supplemental Table 1**). All bacterial strains were grown in brain heart infusion (BHI) broth (Oxoid, United Kingdom) at 30°C overnight without shaking unless stated otherwise.

**Table 1:** Strains used in the study.

Strain	Relevant features	Reference/source
*S. aureus* LMGT 3258	MSSA used in screening	LMGT collection (Ås, Norway)
*S. aureus* Xen 31	A derivative of MRSA ATCC33591 expressing luciferase, used in synergy assay and mouse skin infection model	(26)
*S. equorum* WS 2733	Producer of MP1	(25)
*S. equorum* KAVA	Producer of MP1	This study

### Sample Collection and Screening for Antimicrobial Activity Against *S. aureus*

Biological samples used for antimicrobial screening were obtained from fermented fruits and vegetables. Twenty-five different fruits and vegetables were purchased at a local shop (Oslo, Norway). Each sample (20-30 g) was cut into small pieces and left for three weeks in about equal volume of water with or without NaCl (1-2% final concentration) at outdoor temperature (between 15 and 25 °C). After the incubation, the liquid fraction of each sample was mixed with glycerol (final concentration 20%) and stored at -80 °C until use.

To screen for microorganisms with antimicrobial activity, a small volume (50 µl) of each sample was first 10-fold-serially diluted in sterile saline, then 50 µl of each dilution was transferred to 5 ml of BHI soft-agar (0.7% w/v agar at 50 °C) and the mixture was plated on a BHI agar plate, obtaining a plating density of 10-1000 CFUs per plate. The plates were then incubated overnight at 30°C before covering the lawn with 5 ml of BHI soft-agar containing ca 10^6^ CFU/mL of the indicator strain *S. aureus* LMGT 3258, a methicillin-susceptible *S. aureus* (MSSA). After a further overnight incubation at 30°C, the colonies surrounded with inhibition zones were selected and streaked on fresh BHI agar plates to obtain single colonies. Antimicrobial producing candidates were reconfirmed by having inhibitory activity toward the indicator strain on agar plate assays. Liquid cultures of the candidate antimicrobial producer strains were mixed with glycerol (20%) and stored at -80 °C until use.

### MP1 Purification and Production

The antimicrobial-producing strain *S. equorum* KAVA was grown for 24 h in 1 L BHI broth at 37°C without shaking. The cells were removed by centrifugation at 10,000 × *g* for 15 min at room temperature. The supernatant was applied to a Resource reverse-phase chromatography (RPC) column (1 ml) (GE Healthcare Biosciences) connected to an ÄKTA purifier system (Amersham Pharmacia Biotech). A linear gradient of isopropanol (Merck) with 0.1% (vol/vol) trifluoroacetic acid (TFA) (buffer B) at a flow rate of 1.0 ml min^−1^ was used for elution. RPC fractions were then tested for antimicrobial activity against *S. aureus* LMGT 3258 before selected fractions were further analyzed by mass spectroscopy (MS).

For comparative analysis of MP1 production, *S. equorum* KAVA and *S. equorum* WS 2733 were grown in 5 ml of BHI medium without shaking at 23°C, 30°C and 37°C for 4 days in order to accumulate the bacteriocin. Since MP1 is known to aggregate on the producer cells (27), the cell pellets obtained after centrifugation were treated with 1 ml of 2-propanol to extract MP1. Filter-sterilized supernatants and cell-extracts were analyzed for antimicrobial activity using a microtiter plate assay as previously described (28). The antimicrobial activity of the samples was expressed in bacteriocin units (BU), defined as the minimum amount of bacteriocin that inhibited at least 50% of growth of the indicator strain (*S. aureus* LMGT 3258) in a 200 μl culture volume.

For large scale purification (the optimized protocol), the selected strain was grown in 2 L of BHI broth at 37°C for 4 days. After centrifugation the supernatant was discarded, the cell pellet was washed with saline and the MP1 extraction was performed with 100 ml of isopropanol (Merck). The extract was diluted 5 times with MiliQ water and applied to a Resource reverse-phase chromatography (RPC) column (3 ml) (GE Healthcare Biosciences) connected to an ÄKTA purifier system (Amersham Pharmacia Biotech). A linear gradient of isopropanol with 0.1% (vol/vol) TFA (buffer B) at a flow rate of 1.0 ml min^−1^ was used for elution. The MP1 concentration and purity were determined by HPLC using a Phenomenex Axia Luna C8 100A column (Phenomenex, Norway). Commercial MP1 (Cayman Chemical) with ≥ 95% purity was used as a HPLC standard. After purification, the MP1 solution was dried at 55°C in a SpeedVac concentrator (SPD2010 Integrated SpeedVac, ThermoFisher Scientific, USA). The MP1 pellet was resuspended in DMSO (Sigma-Aldrich) to 1 - 10 mg/ml concentrations and stored at -20 °C before use.

### MS Analysis

MS data were acquired on an Ultraflex MALDI-TOF/TOF (Bruker Daltonics, Bremen, Germany) operated in reflection mode with delayed extraction. Ions of positive charge in the *m/z* range of 200 to 6,000 were analyzed using 25 kV acceleration voltage. The sample spectra were calibrated externally with a calibration standard covering the *m/z* range from 700 to 3,100 (Bruker Daltonics, Bremen, Germany).

### DNA Sequencing

For 16S rRNA gene sequencing, DNA from the isolates with antibacterial activity was isolated by using FastPrep Bio101 (Savant Instruments, USA) and DNA minikit (Omega Bio-Tek Inc., GA), according to the manufacturer instructions. Amplification of the 16S rRNA gene by PCR was carried out using the primers 5F (5′-GGTTACCTTGTTACGACTT-3′) and 11R (5′-TAACACATGCAAGTCGAACG-3′) as previously described (29). PCR products was purified with NucleoSpin Extract II (Macherey-Nagel, Düren, Germany) according to the manufacturer instructions and sent to GATC Biotech, Germany, for sequencing.

Whole genome sequencing (WGS) was performed as described previously described (30). Briefly, genomic DNA was extracted from 1 mL of overnight culture using Qiagen DNeasy Blood & Tissue Kit (Qiagen, Hilden, Germany). DNA libraries were made using the Nextera XT DNA Sample Prep kit (Illumina, San Diego, California, USA) according to the manufacturer instructions. The library was sequenced on Illumina MiSeq platform (Illumina, San Diego, California, USA). Raw Illumina reads were trimmed with Trimmomatic v0.39 (31) to remove the sequencing adapters, quality filtered (Q>20) and *de novo* assembled using SPAdes (v3.7.1) (32). Contigs shorter than 1000 bp or with < 5 times coverage were removed from each assembly prior to gene annotation. The genomes were annotated using the Prokka pipeline (33). WGS data are publicly available at NCBI (GenBank submission ID 2428870). The gene cluster features were edited in a genome browser Artemis (v18.0.0) (34). The linear comparison of gene cluster was created in a Python application, Easyfig (v2.2.2) (35).

### Synergy Assessment

For the assessment of synergistic interactions with MP1, antibiotics with different modes of action and high purity (≥ 97%) were purchased from Sigma-Aldrich. The selected antibiotics were gentamicin, streptomycin, kanamycin, erythromycin, chloramphenicol, tetracycline, penicillin G, fusidic acid and rifampicin. Synergy testing was done with a microtiter plate checkerboard assay as previously described (36). Briefly, equal amount of MP1 was applied on microtiter plate 1 in wells A1-H1 and then diluted two-fold to wells 2-11. Similarly, equal amount of antimicrobial B was applied on microtiter plate 2 in wells A1-A12 and diluted two-fold in wells B-G. Volumes of 50 µl of MP1 from each well of microtiter plate 1 were transferred into microtiter plate 3, except for wells A1-H1. Similarly, the same amounts of antimicrobial B were transferred from microtiter plate 2 into plate 3, except for wells H1-H12 (**Supplemental Figure 1**). Subsequently, an overnight culture of *S. aureus* Xen31 was diluted 25 times prior transferring 100 µl aliquots of the bacterial suspension into each well of plate 3. Wells H2-H12 and A1-G1 were used to estimate MIC values of each antimicrobial alone. The fractional inhibition concentration, was used to define the synergy between antimicrobial A (MP1) and B. FIC values were calculated as follows: FIC = FICa + FICb, where the FICa is the MIC of A in combination/MIC of A alone and FICb is the MIC of B in combination/MIC of B alone. Effects were considered as synergistic if FIC was ≤0.5 (37). MIC values were determined in accordance with CLSI/EUCAST recommendations (https://eucast.org/ast_of_bacteria/guidance_documents/).

### Selection of Suitable Antimicrobial Vehicles

Due to their poor solubility, we performed a search for a suitable vehicle to deliver rifampicin at a concentration of 0.15 mg/ml and MP1 at 0.01 mg/ml; these concentrations were the final concentrations used in the combinatorial topical treatment in mice. Stock DMSO solutions of rifampicin (30 mg/ml) and MP1 (1 mg/ml) were tested for their solubility against a panel of commercially available skin creams with different fat concentrations (22%, 30%, 47%, 60% and 70%), by diluting the stock solutions 1:65 into each cream. The mixture was heated to 50°C to reduce viscosity, mixed vigorously on a vortex for 5 min and then centrifuged for 15 min at 15000 xg at room temperature. High solubility was reached when no visible pellet was seen at the bottom of the tubes. Based on the levels of antimicrobial solubility, APO base 30% cream (Teva, Finland) was found the most suitable and was selected as the antimicrobial vehicle for all *in vivo* experiments in this study. The mixture containing 0.15 mg/ml rifampicin and 0.01 mg/ml MP1 in APO base 30% cream did not lose its antimicrobial activity after a two-week storing at 5°C and was chosen for *in vivo* experiment.

### Murine Experiments

Experiments on mice were approved by the Norwegian Food Safety Authority (Oslo, Norway), application no. 20/10793. In total, 39 female BALB/cJRj mice of four weeks of age were purchased from Janvier (Le Genest-Saint-Isle, France). Three to four mice were housed per cage during the whole experiment and maintained on a 12-hour light/12-hour dark cycle with *ad libitum* access to water and a regular chow diet (RM1; SDS Diet, Essex, UK). Mice were acclimatized in our mouse facilities for two weeks before the start of the experiments; hence the age of mice at the start of the experiments was six weeks.

Before infection and treatment, the mice were shaved as follows: mice were anesthetized with Zoletyl Forte, Rompun, Fentadon (ZRF) cocktail (containing 3.3 mg Zoletil forte, 0.5 mg Rompun and 2.6 µg Fentanyl per 1 ml 0.9% NaCl) by intraperitoneal injection (0.1 ml ZRF/10 g body weight) and shaved on the back and flanks with an electric razor. The remaining hair was removed by hair removal cream (Veet, Reckitt Benckiser, Slough, UK) according to the manufacturer’s instructions. The next day the mice were again anesthetized with ZRF cocktail (0.1 ml/10 g body weight) and two skin wounds were made on the back of every mouse with a sterile biopsy punch 6 mm in diameter (Dermal Biopsy Punch, Miltex Inc, Bethpage, NY). Prior to infection, overnight-grown *S. aureus* Xen31 cells were washed twice in sterile saline and then suspended in ice-cold PBS buffer. Each wound was inoculated with 10 μl of PBS containing ca 2x10^7^ CFU of *S. aureus* Xen31 cells using a pipette tip. After bacterial application the mice were kept on a warm pad for 10-15 min to dry the inoculum and the wounds were then covered with a 4×5 cm Tegaderm film (3M Medical Products, St. Paul, MN, USA). Mice were then left for 24h for the infection to establish. The day after (24 h post infection; PI) the mice were anesthetized with 2% isoflurane and the luminescent signal was measured by IVIS Lumina II, Perkin Elmer (2 min exposure time). The luminescent signal was quantified by the software Living Image (Perkin Elmer) from regions of interest (ROIs) around the wound and expressed as photons/second/cm^2^/steradian.

From this point, the mice were divided into 5 groups and subjected to 5 different treatments: one treated with MP1 (10 µg/ml) in APO base 30% cream (n=8), one treated with rifampicin (0.15 mg/ml) in APO base 30% cream (n=8), one treated with the mixture of MP1 and rifampicin (0.15 mg/ml rifampicin and 10 µg/ml MP1, in APO base 30% cream, n=8), one treated with the vehicle (APO base 30% cream) as a negative control (n=8), and one treated with fucidin cream (2% fusidic acid in a cream base; LEO Pharma, Denmark) as a positive control (n=7). All treatments were performed once a day. To assess if the treatment had a long-lasting effect, 4 mice from each group received the treatment for 4 days and were left untreated in a separate cage until the end of experiment while the remaining 4 mice (3 mice for the fucidin group) continued to receive treatments once a day until the end of the experiment (9 days). In all treatment groups, 50 µl of either antibacterial solution or control substance was injected into each wound under the Tegaderm using an insulin syringe (BD SafetyGlide™; 29G needle). The bioluminescent signal, produced by *S. aureus* Xen31 luciferase was recorded once per day before each treatment, during the entire course of the experiments. At the end of each experiment mice were euthanized by cervical dislocation.

### Statistical Analysis

All *in vitro* assays were performed three times. For statistical analyses and graphs, R Studio (version 1.0.15; https://rstudio.com/products/rstudio/download/) was used.

## Results

### Screening for Bacteriocin Producers

Twenty-five fruit and vegetable samples were used as source for screening of bacteria with antimicrobial activity against *S. aureus* LMG3258. Since nisin producers are frequently found in such samples (38) and we wanted to exclude these from the current screen, the isolates with activity against *S. aureus* LMG3258 were re-tested against the nisin-immune strain LMGT 2122 (a known nisin producer). Most of the isolates with activity against LMG3258 were indeed nisin producers (data not shown). However, one isolate, called KAVA, inhibited both LMG3258 and LMGT 2122, and was therefore chosen for further analysis. By 16S rRNA genotyping, the isolate KAVA was found to be *Staphylococcus equorum* (hereafter called *S. equorum* KAVA).

### *S. equorum* KAVA Produces Micrococcin P1

**Figure 1:**
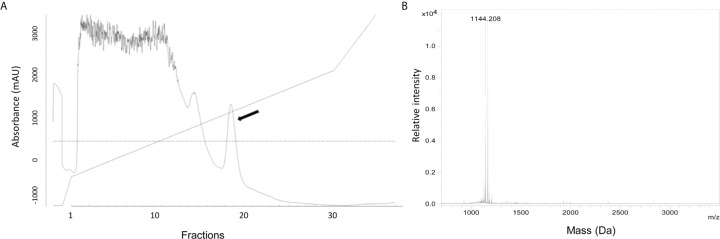
**(A)** RPC elution profile of MP1 from the cell-free supernatant of *S. equorum* KAVA. MP1 elution peak indicated with an arrow. **(B)** Mass spectrometry analysis of the active fraction of MP1 after the RPC. The inset is amplification of the MP1 peak area.

To define the nature of the active substance, it was purified from the culture supernatant of *S. equorum* KAVA. The substance was eluted with 45% 2-propanol/TFA during PRC, indicating that the antimicrobial molecule was relatively hydrophobic (**Figure 1A**). MS analysis showed that the antimicrobial substance had a mass of 1144 Da (**Figure 1B**), a size corresponding to the known antimicrobial peptide micrococcin P1 (MP1). To prove the identity of the active substance further, we tested the purified fractions of *S. equorum* KAVA against a panel of 30 different bacterial species. As expected, the substance produced by *S. equorum* KAVA was active only against Gram-positive bacteria, including *Listeria* spp, enterococci, staphylococci, but not against Gram-negative bacteria such as *Escherichia coli*, *Acinetobacter baumanii* and *Pseudomonas aeruginosa* (**Supplemental Table 1**). Such activity spectrum is in line with published results for MP1 (22).

### Whole Genome Sequencing of *S. equorum* Confirms the Presence of Novel MP1 Gene Clusters

**Figure 2:**
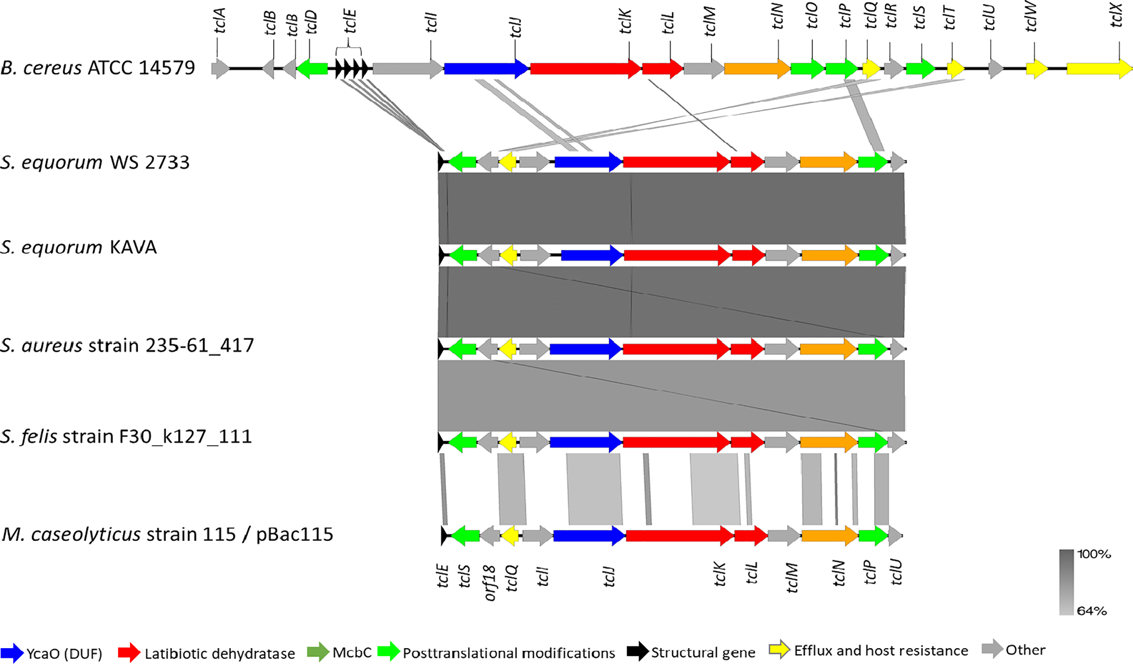
Genetic organization of micrococcin P1 gene cluster of *S. equorum* WS 2733 and *S. equorum* KAVA in comparison with reference gene cluster in the plasmid pBac115 of *M. caseolyticus* (accession number: KM613043), *S. aureus* strain UP 1591 plasmid unnamed (GenBank accession: CP047810), *S. aureus* strain 235-61_417 plasmid unnamed (GenBank accession: VUGU01000042.1) and *S*. *felis* strain F30_k127_111 (GenBank accession:QKYH01000057). The open reading frames that are involved in micrococcin P1 production are shown in different colors. Areas shaded in grey indicate homologous regions with 69-100% nucleotide identity.

To corroborate our finding further, whole genome sequencing of *S. equorum* KAVA and the strain *S. equorum* WS 2733, a known MP1 producer isolated from cheese (25), were performed, and indeed two very similar MP1 gene clusters were found (**Figure 2**). Interestingly, beside these two genomes, a database search led to the identification of similar MP1 gene clusters in other staphylococcal and non-staphylococcal genomes: one in a SSTI-associated *S. aureus* isolate (accession number VUGU01000042.1), one in a *S. felis* strain isolated from an otitis infection in a cat (accession number QKYH01000057.1), one in a *Bacillus cereus* strain (accession number NZ_CP034551.1), and one on the plasmid pBac115 (accession number: KM613043) from *Macrococcus caseolyticus*.

MP1 gene clusters from *S. equorum* KAVA and *S. equorum* WS 2733 turn out to be almost identical to each other and both were similar to the pBac115 cluster (39) and other MP1 clusters from staphylococci (**Figure 2**). All staphylococcal MP1 gene clusters consisted of 12 genes and possessed only one copy of the MP1 structural gene (*tclE*) and a single immunity gene (*tclQ*), while MP1 gene cluster of *B. cereus* ATCC 14579 contained four copies of *tclE* and two copies of immunity genes *tclT* and *tclQ* (**Figure 2**). Using EMBOSS Needle pairwise sequence alignment (40) we found that the most conserved proteins across the clusters were the MP1 structural peptide, and its immunity protein (**Table 2**). Other proteins with high similarity were TclJ and TclN (which together catalyze the conversion of all six cysteines in the MP1 backbone to thiazole rings), TclK, TclL (Ser/Thr dehydration), TclM and TclP (unknown function) (41). The genes *tclA*, *tclB, tclD, tclO, tclX* were present only in *B. cereus* ATCC 14579 gene cluster. The staphylococcal MP1 clusters had only one gene with no homology in *B. cereus* ATCC 14579 gene cluster, namely *orf18*, encoding a 160 amino acid residue protein with unknown function (42) (**Figure 2**).

**Table 2:** Similarity/identity score of core proteins involved in the MP1 production in different strains in comparison with reference MP1 cluster in *B. cereus* ATCC14579 (*).

	TclE	TclI	TclJ	TclK	TclL	TclM	TclN	TclP	TclT
*M. caseolyticus*	65.4/51.9	20.0/12.1	43.9/30.0	46.5/27.8	42.2/26.7	43.3/26.1	41.4/25.2	54.7/36.3	78.7/59.6
*S. equorum* KAVA	67.3/55.8	20.0/11.1	39.2/26.7	45.5/26.2	46.8/28.3	38.4/21.5	41.8/24.9	57.0/41.9	78.0/57.4
*S. equorum* WS 2733	67.3/55.8	22.2/14.2	41.8/27.4	45.8/26.8	46.2/28.3	38.0/25.1	39.0/22.9	58.6/42.6	78.0/57.4

*The pairwise sequence alignment was made with EMBOSS Needle Pairwise Sequence Alignment Tool (https://www.ebi.ac.uk/Tools/psa/emboss_needle/).

### Comparison of MP1 Production by *S. equorum* Strains

Since *S. equorum* KAVA and *S. equorum* WS 2733 were readily available, it was of interest to investigate which one had the highest MP1 production ability. To address this point, we compared their growth and bacteriocin production profiles in BHI medium at different temperatures (23°C, 30°C and 37°C). Although both strains grew equally at the three temperatures (data not shown), their bacteriocin production was different. As can be seen in **Table 3**, *S. equorum* KAVA produced 80 BU/ml at the two lower temperatures, 23°C and 30°C, and 160 BU/ml at 37°C, while *S. equorum* WS 2733 produced 40-80 BU/ml at the two lower temperatures but, surprisingly, no or poor production was detected at 37°C (**Table 3** and **Supplemental Figure 2**).

**Table 3:** Comparison of MP1 production (in BU/ml) by *S. equorum* KAVA and *S. equorum* WS 2733 at different temperatures.

	Supernatant (BU/ml)			Cell extract (BU/ml)		
	23°C	30°C	37°C	23°C	30°C	37°C
*S. equorum* WS 2733	40	80	0	160	2500	0
*S. equorum* KAVA	80	80	160	1200	2500	2500

As bacteriocins of the thiopeptide family are often adsorbed on the producer cells due to their high hydrophobicity (43), cell pellets from the two producers were also obtained and treated with equal volumes of 2-propanol to extract the bacteriocin into the organic phase. As expected, the organic fractions displayed the highest activity; the increase was 4-30-fold for *S. equorum* WS 2733 and 15-30-fold for *S. equorum* KAVA, compared to their respective water-soluble fractions depending on the growth temperature conditions (**Table 3**). Notably, extracts from *S. equorum* WS 2733 grown at 37 °C retained no bacteriocin activity.

Given that the extraction of the bacteriocin from *S. equorum* KAVA cells gave the highest yields, we used this strain as a main source of MP1 for further studies. By modulating the growth conditions (BHI broth, 37°C, four-day incubation), we optimized the protocol (see Materials and Methods) and were able to purify MP1 at a concentration of 15 mg/L of broth with a 98% purity as estimated by RPC HPLC (**Supplemental Figure 3**).

### Search for Synergistic Antimicrobial Activities

MP1 is a peptide with high antimicrobial activity against many Gram-positive bacteria. However, sensitive bacteria can easily become resistant to MP1 by single-point mutations within the gene encoding the L11 ribosomal protein (44), making this antimicrobial less viable in therapeutics. This was also confirmed in recent works against MRSA (45, 46) where we observed numerous MP1-resistant mutants in our antimicrobial sensitivity assays. To avoid this resistance problem, we searched for antimicrobials which could act synergistically with MP1. Nine antibiotics of different classes and with different modes of action were chosen for the synergy experiment (**Table 4**). Indeed, by using the checkerboard assay, synergistic effects against the strain MRSA Xen31 were found between MP1 and the following antibiotics: tetracycline, penicillin G, chloramphenicol, fusidic acid and especially with rifampicin (**Table 4**). Fractional inhibitory concentration (FICs) values for tetracycline, penicillin G, chloramphenicol and fusidic acid in combination with MP1 were between 0.13 to 0.18. Most notably, the combination with rifampicin reduced MIC values from >100 µg/ml to 1.5 µg/ml for the antibiotic and from 2.5 µg/ml to 0.1 µg/ml for MP1, resulting in a FIC value equal to 0.05 (FIC values ≤0.5 are considered synergistic between two components) (37). Based on these results we sought to further explore the therapeutic potential of the combinatory effect of MP1 and rifampicin in a murine infection model (see below).

**Table 4:** Synergy assessment between MP1 and a panel of antibiotics against MRSA Xen31.

	Single antimicrobial (µg/ml)	Comb. with MP1Antibiotic/MP1, (µg/ml)	FIC*
MP1	2.5	–	
Gentamicin	>250	4/2.5	1.0
Streptomycin	>250	125/1.25	1.0
Kanamycin	>250	125/1.25	1.0
Erythromycin	>250	125/1.25	1.0
Chloramphenicol	62	4/0.3	0.18
Tetracycline	150	4.5/0.3	0.15
Penicillin G	>2500	16/0.16	0.13
Fusidic acid	0.6	0.04/0.16	0.13
Rifampicin	>100	1.5/0.1	0.05

*Antimicrobial combinations are considered synergetic if fractional inhibition concentration is ≤0.5 (37).

### Choosing the Vehicle for the Antimicrobials

MP1 and rifampicin were dissolved in the hand cream Apo Base with 30% fat (Teva, Finland), which was the most suitable vehicle in terms of solubility and appropriate viscosity (see Materials and Methods). For topical use in mice the final mixture in APO Base 30 cream contained 10 µg/ml MP1 and 150 µg/ml rifampicin which were about 100 times higher than their MIC values recorded in the checkerboard assay, since MRSA Xen31 parental strain *S. aureus* ATCC 33591 is known to produce biofilms on surfaces within 24h (47) and staphylococcal biofilms are 10- to 1000-fold more resistant to antimicrobials compared to planktonic cells (48).

**Figure 3:**
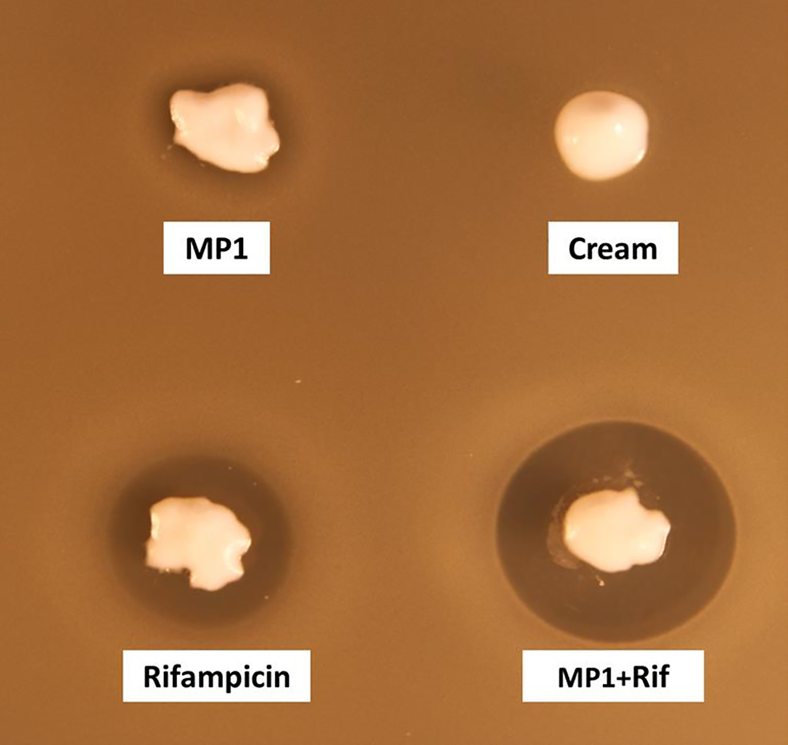
Assessment of the antimicrobial activity of MP1(10 µg/ml), rifampicin (150 µg/ml) and the combination (MP1, 10µg/ml + rifampicin, 150µg/ml) in APO base cream 30%. Cream with not addition was included as negative control. The activity was tested with softagar overlay assay using MRSA Xen31 as indicator strain.

The cream by itself did not inhibit MRSA while the cream containing the antimicrobials, hereafter referred to as the MP1-rifampicin mixture, displayed strong antimicrobial activity as expected (**Figure 3**).

### The MP1-Rifampicin Mixture Is Effective Against MRSA in a Murine Skin Wound Infection Model

**Figure 4:**
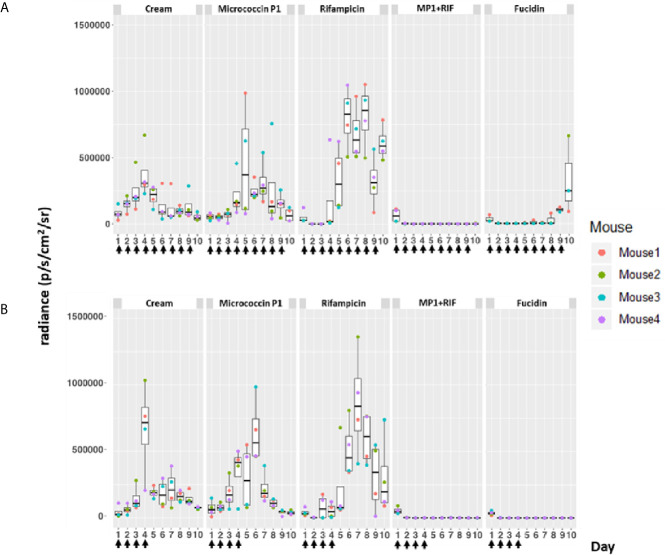
Bioluminescence from mice skin infections during different treatments. Box plots of bioluminescent signals produced by MRSA Xen31 (in photons per second per square centimeter per steradian) in differently treated mouse groups. The days of treatment are indicated with arrows. **(A)** mice received daily treatments for nine days. **(B)** mice received daily treatments for four days before they were left untreated for the rest of the experiment. The area within each box represents the interquartile region (IQR), which comprises the second and third quartiles and describes the interval of values where the middle 50% of the observed data are distributed. The horizontal black line within each box represents the median value. The extent of the IQR (box height) express the degree of variability measured within the middle 50% of the observed data, with whiskers extending out at either side of the boxes marking the minimum and maximum observed values, as well as the variability outside the middle 50% of values (whisker length). Outliers are displayed as data that extend out of the whisker limit (1.5 times the IQR).

**Figure 5:**
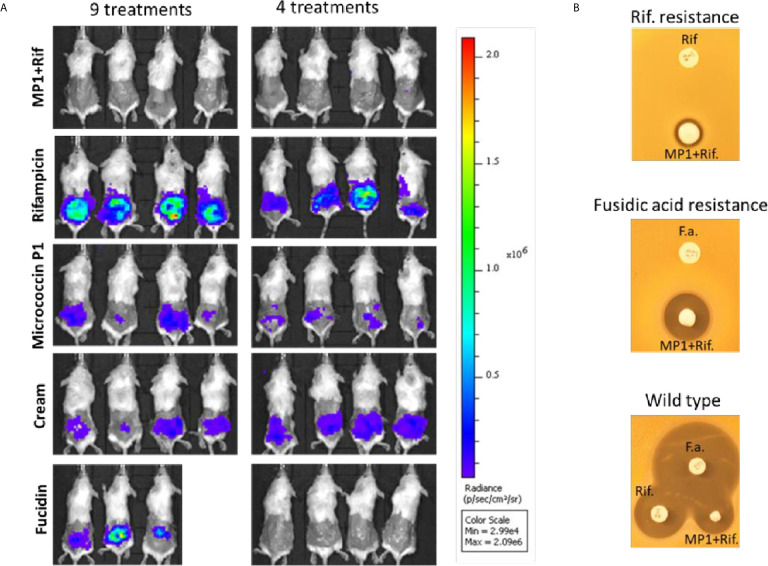
**(A)** *In vivo* images of bioluminescent signals produced by MRSA Xen31(in photons/second/cm^2^/steradian) from the different mouse groups on the last day of the experiment; left column: with nine daily treatments; right column with four daily treatments. **(B)** Rifampicin and fucidin resistance develops during the treatment of mice. Rifampicin resistant cells (Rif-resistance) and fucidic acid resistant cells (Fucidic acid resistance) isolated from the wounds with strong bioluminescent signals were rechallenged and shown to be resistant to rifampicin (Rif) and fusidic acid (F.a.) but not to the MP1-rifampicin mixture. Wildtype MRSA Xen31 cells exposed to the MP1-ripamicin mixture, rifampicin and fusidic acid were sensitive to all three antimicrobials.

In order to validate the therapeutic value of the MP1-rifampicin mixture *in vivo*, we used a recently established murine skin wound infection model (45). This model applies the luciferase-expressing *S. aureus* Xen31, a derivative of MRSA ATCC33591 (PerkinElmer). This strain allows us to monitor the bacterial growth-dependent luminescence intensity which is proportional to the growth of *S. aureus* Xen31 (49) during the entire course of the experiment. Mice were divided into five groups (n=8 per group except in one group where n=7), for different treatments: MP1 (group 1), rifampicin (group 2), vehicle (Apo Base 30 cream) alone as negative control (group 3), MP1-rifampicin mixture (group 4) and fucidin cream (group 5) as positive control. Fucidin cream contains 20 mg/ml of fusidic acid and commonly used against MRSA skin infections (50). Wounds on the back of each animal were infected with *S. aureus* Xen31 (approximately 2x10^7^ CFU/wound), covered with Tegaderm (a transparent wound dressing) and the infection was allowed to establish for 24 h prior beginning each treatment regimen. In addition, each group was divided into two subgroups where one subgroup received daily treatments from day 2 until the end of the experiment (**Figures 4A** and **5A**) while the other subgroup was treated only four times during first four days and after that left untreated until the end of the experiment, in order to examine the long-term effect of the treatments (**Figures 4B** and **5B**).

As can be seen in **Figure 4**, the bioluminescent signals from all mice wounds were clearly visible prior to all treatments (day 1 post infection; PI). Consistent with the *in vitro* results, the bioluminescent signals generated in the wounds treated with the vehicle (pure APO-base cream) displayed a steady increase from day 2 PI and peaked between day 4 and 7 PI in all mice. As expected, the application of MP1 alone (10 µg/ml) was not able to stop the MRSA wound infection in all mice throughout the experiment. In rifampicin-treated mice (150 µg/ml) the bioluminescent signals sharply declined the first day after treatment in all mice (day 2 PI), however, the next day (day 3 PI), two mice displayed high luminescence, and by day 5 PI this was the case for all rifampicin-treated mice, likely due to the selection of rifampicin-resistant MRSA cells within the wounds (**Figure 4B**). In contrast, in mice treated with the MP1-rifampicin mixture, the luminescent signals not only declined abruptly within 24 hours after the first treatment but stayed below the detection limit over the entire period of the experiment (10 days). Notably, for the four mice which received only four treatments of MP1-rifampicin mixture, no re-infection or recurrence appeared during the rest of the experiment, indicating that the antimicrobial effect of the MP1-rifampicin combination lasted for at least 5 days after four treatments (**Figure 4B**). Fucidin was used as a positive control, and, as expected, caused a sharp reduction of luminescent signals within 24 hours after the first treatment. However, on day 6 PI one mouse from the group which received the treatment every day had a slight increase of the luminescent signals (**Figure 4A**). Next day (Day 7 PI) the signal could not be detected but appeared again on day 8 PI. On day 10, all three mice had high luminescent signals despite being treated with fucidin every day (**Figures 4A** and **5A**). Interestingly, for mice received only 4 fucidin treatments and then left untreated for 5 days, no signs of re-infection were detected after the fourth treatment (**Figures 4B** and **5A**).

The results from the rifampicin and fucidin treatments suggested resistance development in the MRSA strain against these antibiotics. In order to confirm this, on the last day of the experiment, luminescent bacteria were isolated from fucidin-treated and rifampicin-treated mice and rechallenged with the antibiotics. As expected, all isolates were indeed resistant to fucidin and rifampicin, respectively, but not to the MP1-rifampicin mixture (**Figure 5B**).

Furthermore, no mice showed any obvious signs of abnormal behavior, neither in the non-treated group nor in the treated groups, indicating that the different treatments had no obvious toxic effects.

## Discussion

The emergence of multidrug-resistant bacteria has been recognized as a major public health problem. One strategy to combat such bacteria is revitalizing old antimicrobials which were discovered in the past but are not much used in today’s medicine because of different reasons, e.g., low or expensive production, lack of delivery means, or a consequence of resistance development (51, 52). Thiopeptides represent a very promising class of neglected antimicrobials. Despite their potent antimicrobial activity thiopeptides have been poorly exploited in therapeutic treatments so far due to the high rate of resistance development, challenging synthesis, poor aqueous solubility and associated low bioavailability (53). In this work we describe a cost-efficient production of the thiopeptide MP1 and show that MP1 in combination with rifampicin had very good synergistic effects on MRSA. Its therapeutic and synergistic properties were successfully validated in a murine skin infection model.

Several attempts have been made to synthesize MP1 chemically to reduce its cost and possibly modify its structure to make it more water-soluble, but so far these synthetic approaches are not scalable and cost-effective (54, 55). To improve the effectiveness of MP1 production, fermentation can be an alternative to synthetic approaches. In this work we isolated a new *S. equorum* strain from a sample of fermented vegetable which displayed high and stable MP1 production. We then propose a very simple and cost-effective method for purification of MP1 from *S. equorum* by extracting MP1 from the producer cells with 2-propanol with subsequent one-step RPC purification of the water-diluted extract. Using this method, we were able to obtain 15 mg of 98% pure MP1 from 1L of BHI medium (**Supplemental Figure 3**).

The difference in temperature dependent MP1 production between the two *S. equorum* strains, suggests that the production is somehow regulated differently. However, the two strains have almost identical MP1 gene clusters (**Figure 2** and **Table 2**), indicating that either a subtle difference within the loci or a difference outside the loci could be the cause for this phenotypic difference. Interestingly, there are major genetic differences between the staphylococcal strains and other MP1 producers. For instance, the staphylococcal MP1 gene cluster comprises 12 genes while it has 24 genes in *B. cereus* ATCC 14579. Furthermore, the MP1 locus contains only one structural gene in the former while four consecutive structural genes are found in the latter. The differences also extend to their final product(s). The staphylococcal strains appear to produce only one product, namely MP1 (39), while *B. cereus* ATCC 14579 produces a mixture of similar thiopeptides with different posttranslational modifications [thiocillin I, II, III, MP1 and micrococcin P2 (MP2)] (41). In terms of purification of MP1 the staphylococcal strains will be preferable because the purified MP1 will not be contaminated with physico-chemically similar thiopeptide species as it would with *B. cereus* ATCC 14579.

No cross-resistance has been reported between common antibiotics and thiopeptides (44), suggesting a possible combinatory approach for therapeutic use. Indeed, using MRSA as a target pathogen, MP1 was found to have synergistic effect with several antibiotics, especially with rifampicin which gave the best synergy (**Table 4**). Rifampicin, also known as rifampin, is a broad-spectrum lipophilic antimicrobial agent that inhibits bacterial RNA polymerase (56). Recently rifampicin has gained much attention due to its bactericidal activity against *S. aureus*, including MRSA (57). Besides its high cellular permeability, rifampicin is one of the few antimicrobial agents that can penetrate biofilms and kill organisms in the sessile phase of growth (58).

Our results, both *in vitro* and *in vivo* showed a clear synergistic effect between MP1 and rifampicin against MRSA Xen31 (**Table 4** and **Figure 3**). While neither MP1 nor rifampicin had any long-lasting therapeutic effect on MRSA Xen31 in the murine model (**Figure 4**), the mixture of MP1 and rifampicin efficiently removed the pathogen from infection sites and prevented its recurrence and resistance development. The combinatory mixture had a long-lasting effect as no obvious sign of the pathogen was seen at least 5 days after the daily 4-day scheme of treatment (**Figure 4**). This is not the case for fusidic acid which is a commonly used antibiotic in treatment of skin infections. Our present study and others’ (59) demonstrate that fusidic acid monotherapy is inefficient in the treatment of staphylococcal infections due to rapid resistance development.

It is still debatable whether the combination of rifampicin with other antimicrobials truly confers additional effectiveness over rifampicin monotherapy in human and animal infections, since animal models show contrasting results (58). For example, in a rat model of chronic subcutaneous staphylococcal foreign-body infection rifampicin was an important adjuvant to vancomycin and fleroxacin (60). The synergy with vancomycin was also demonstrated on human patients against MRSA septicemia in burns (61), in a knee prosthetic infection model (62) and in the treatment of nosocomial MRSA induced pneumonia (63). In addition to these examples, several other studies using different animal models and/or other types of infection have also shown good synergistic effects of rifampicin with other antibiotics such as linezolid (64), β-lactams or glycopeptides (65), and some topical antimicrobials (66).Yet, other results provided no overall benefit of the adjunctive effect of rifampicin over standard antibiotic therapy against *S. aureus*; neither *in vitro* (67, 68). In addition, the use of rifampicin adjunctive therapy for the treatment of SSTI is not recommended by the Infectious Diseases Society of America (69). These studies with contrasting outcomes highlight a complexity of the types of diseases, the hosts and the combinatory antibiotics in question. Thus, the synergistic properties of rifampicin as therapeutic option should be evaluated with great care to avoid inefficiency or other potential collateral effects before being used in the intended hosts.

Topical treatment often allows the use of relatively high concentrations of antimicrobials at the wound sites compared to systemic treatment (70). However, too high antimicrobial concentrations may cause cytotoxic effects on skin cells and prevent rapid wound healing (71). One possible solution to avoid this is to use synergistic antimicrobial combinations as shown here for MP1-rifampicin. Such an approach also provides an effective mean to prevent resistance development. Nevertheless, further research is needed to unravel the molecular mechanisms underlying the synergistic effects, including the influence on mutation rates, how bacterial cells respond to sublethal antimicrobial concentrations and the mechanism behind the bactericidal effect. Such knowledge will help design safer and more efficient drugs before testing them in clinical settings.

## Data Availability Statement

The datasets presented in this study can be found in online repositories. The names of the repository/repositories and accession number(s) can be found below: https://www.ncbi.nlm.nih.gov/genbank/, submission ID 2428870.

## Ethics Statement

The animal study was reviewed and approved by Norwegian Food Safety Authority (Oslo, Norway), application no. 20/10793.

## Author Contributions

KO: manuscript writing, *in vitro* and *in vivo* synergy experiments, MP1 purification. CK: manuscript writing, statistical analysis. AT: WGS, in silico DNA analysis. MK: manuscript writing. TT: funding, conceptualization. SS: manuscript writing, bacterial strain providing. HC: mouse model supervision. DD: project supervision. All authors contributed to the article and approved the submitted version.

## Funding

This work was supported by the research council of Norway, from the program Forny (project no. 296220), and the program Better Health and Quality of Life (project No. 273646).

## Conflict of Interest

The authors declare that the research was conducted in the absence of any commercial or financial relationships that could be construed as a potential conflict of interest.

## Supplementary Material

Supplementary Figure 1 Microtiter plate checkerboard assay scheme. See description in Materials and Methods.

Supplementary Figure 2 MP1 production by *S. equorum* WS 2733 and *S. equorum* KAVA at different temperatures.

Supplementary Figure 3 RPC elution profile of MP1 from diluted 2-propanol cell-extract of *S. equorum* KAVA. B. RPC HPLC purity analysis of the active fractions from the purification. MP1 elution peaks are indicated with arrows.

Supplementary Table 1 The inhibitory spectrum of the purified fractions of *S. equorum* KAVA.
