# Supplementary figures and images for: A Strong Synergy Between the Thiopeptide Bacteriocin Micrococcin P1 and Rifampicin Against MRSA in a Murine Skin Infection Model
Source: Front Immunol. 2021 Jul 2;12:676534. doi: 10.3389/fimmu.2021.676534 (PMC8284338; doi:10.3389/fimmu.2021.676534)

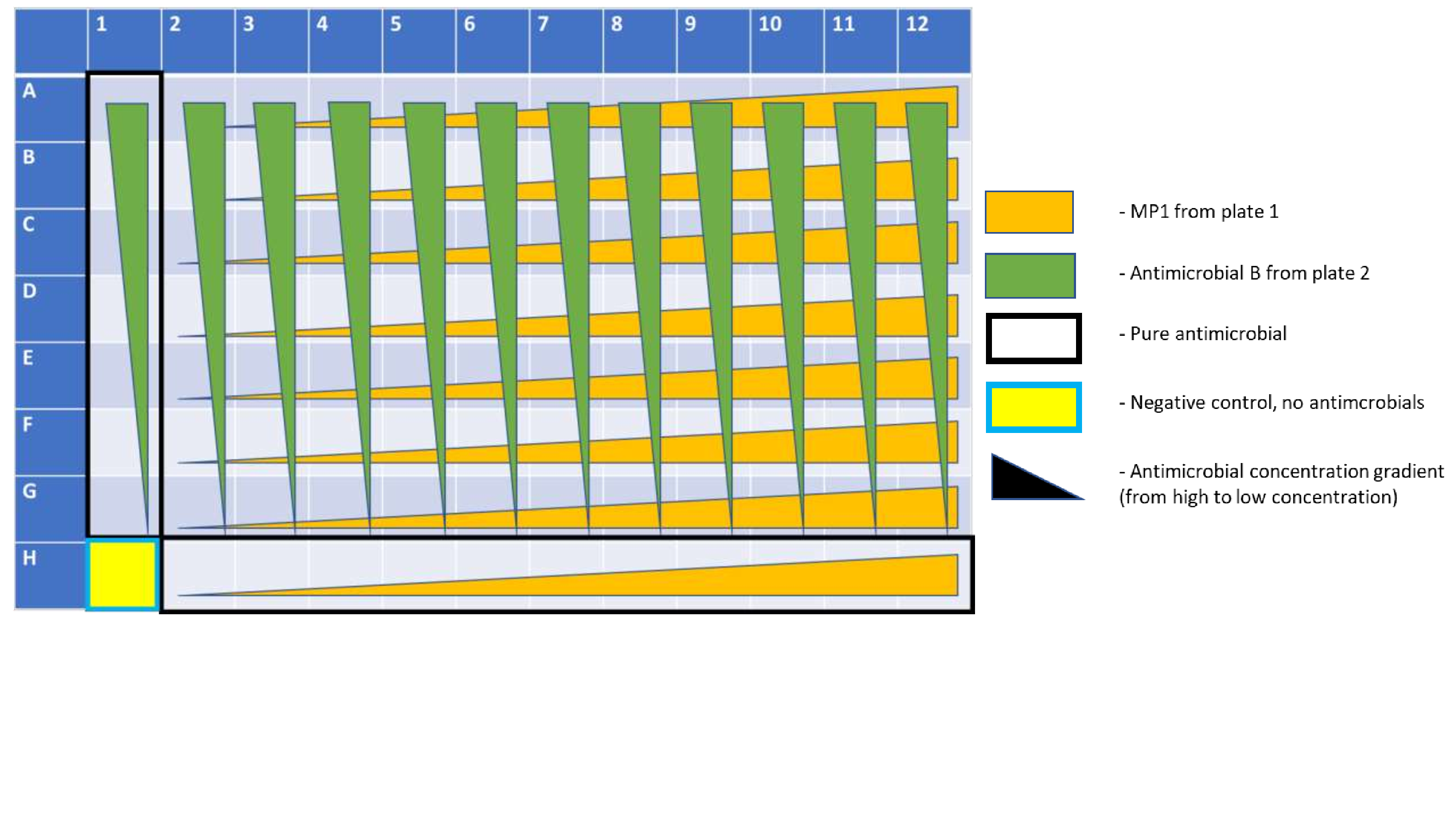

Supplement: Supplementary Figure 1 — Microtiter plate checkerboard assay scheme. See description in Materials and Methods. [file Image_1.tiff]

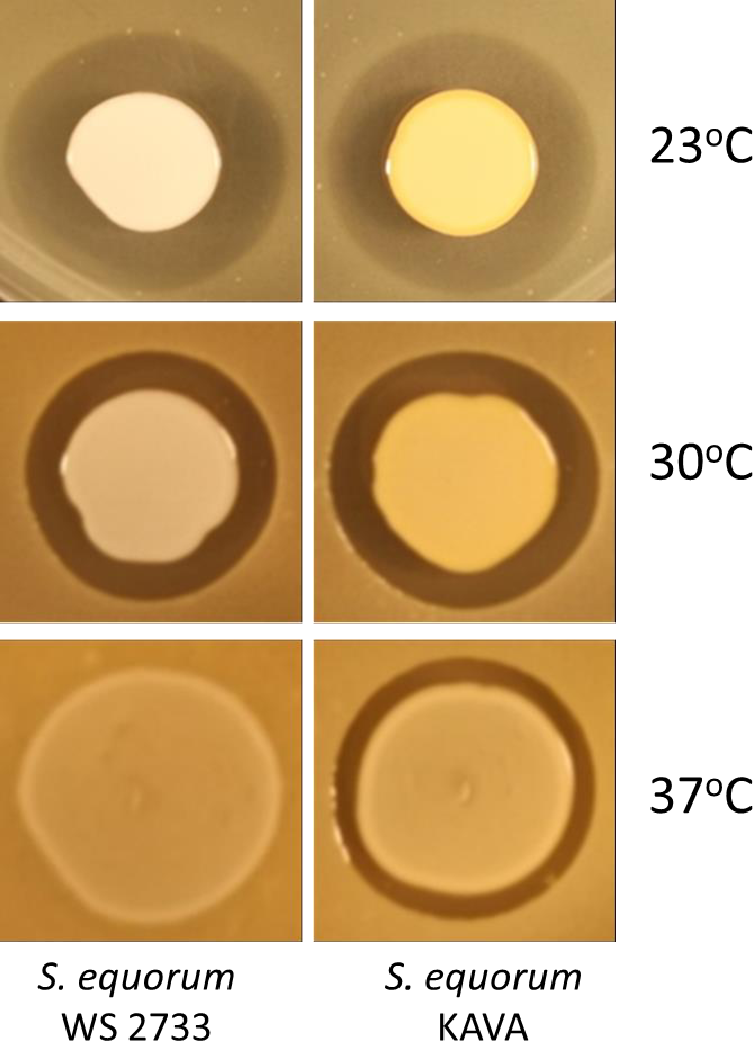

Supplement: Supplementary Figure 2 — MP1 production by S. equorum WS 2733 and S. equorum KAVA at different temperatures. [file Image_2.tif]

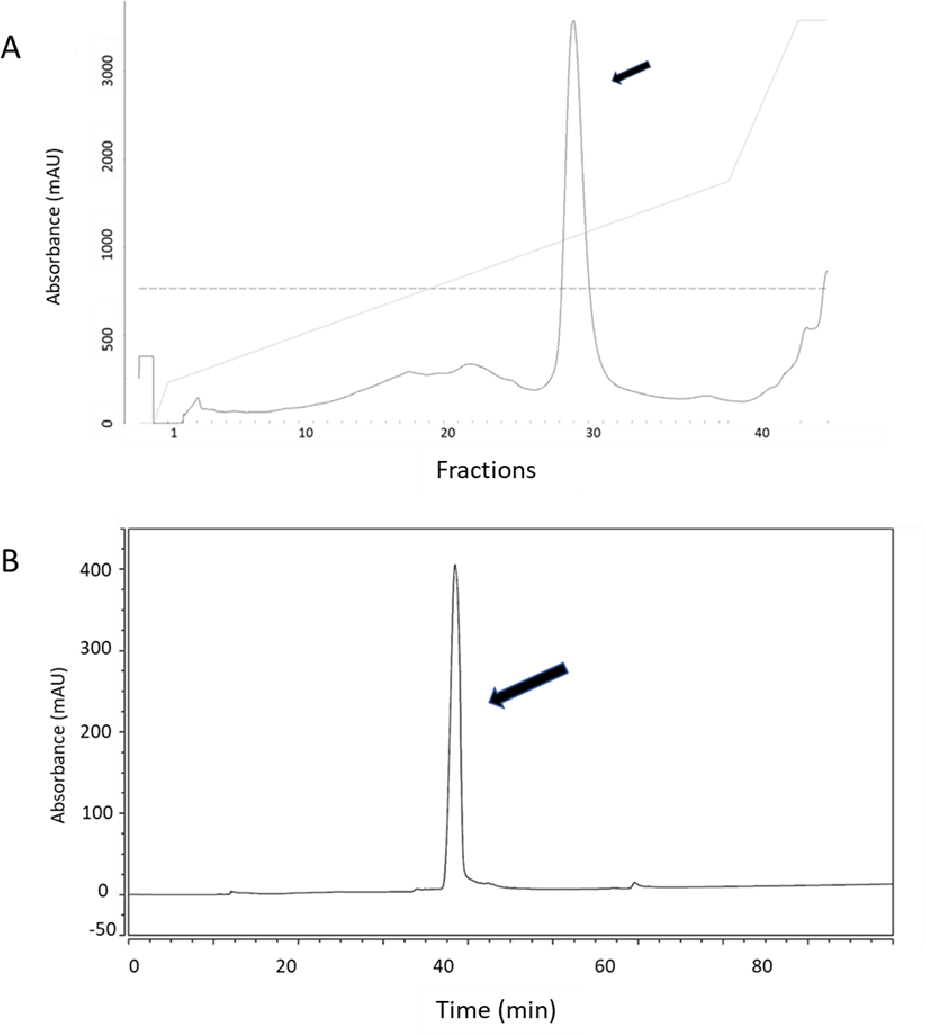

Supplement: Supplementary Figure 3 — RPC elution profile of MP1 from diluted 2-propanol cell-extract of S. equorum KAVA. B. RPC HPLC purity analysis of the active fractions from the purification. MP1 elution peaks are indicated with arrows. [file Image_3.tif]
