# Supplementary material for: A Strong Synergy Between the Thiopeptide Bacteriocin Micrococcin P1 and Rifampicin Against MRSA in a Murine Skin Infection Model
Source: Front Immunol. 2021 Jul 2;12:676534. doi: 10.3389/fimmu.2021.676534 (PMC8284338; doi:10.3389/fimmu.2021.676534)
Supplement: Supplementary Table 1 — The inhibitory spectrum of the purified fractions of S. equorum KAVA. [file Table_1.docx]

**Supplemental Table 1**

The inhibitory spectrum of the purified fractions of *S. equorum* KAVA

| Indicator strain | activity |
| --- | --- |
| *Bacillus cereus* 25 LMGT 2735 | + |
| *B. cereus* LMGT 2805 | ++ |
| *E. avium* 208 LMGT 3465 | ++ |
| *E. faecalis* LMGT 2003 | ++ |
| *E. faecalis* UI50 LMGT 2333 | + |
| *E. faecium* AL41 LMGT 2876 | ++ |
| *E. faecium* LMGT 2787 | + |
| *Lactobacillus helveticus* ATCC 15009 B LMGT 2717 | ++ |
| *L. plantarum* 32 ACA-DC 3333 LMGT 3215 | ++ |
| *L. plantarum* C11 LMGT 2358 | ++ |
| *L. plantarum* ssp 965 LMGT 2003 | ++ |
| *L. sakei* 148 LMGT 2361 | ++ |
| *L. sakei* 706 LMGT 2334 | ++ |
| *Lactococcus garvieae* DCC43 LMGT 3390 | ++ |
| *L. lactis* F14 LMGT 2081 | ++ |
| *L. lactis* IL1403 LMGT 2705 | ++ |
| *L. lactis* NCDO 1403 Nisin LMGT 2122 | ++ |
| *Lactococcus* strain F4-13 LMGT 2070 | ++ |
| *Listeria innocua* LMGT 2710 | ++ |
| *L. innocua* LMGT 2785 | ++ |
| *L. monocytogenes* 279 serotype 4 LMGT 2650 | ++ |
| *L. monocytogenes* 400 serotype 4 LMGT 2651 | ++ |
| *L. monocytogenes* LMGT 2652 | ++ |
| *Pediococcus pentosaceus* FBB 61.1 LMGT 2001 | ++ |
| *P. pentosaceus* FBB 63 B LMGT 2722 | ++ |
| *Staphylococcus aureus* LMGT 3022 | + |
| *S. aureus* LMGT 3023 | + |
| *S. aureus* LMGT 3242 | ++ |
| *S. aureus* LMGT 4089 | + |
| *S. aureus* MRSA Fra UiBonn 2005 LMGT 3266 | ++ |
| *Leuconostoc gelidum* *LMGT 2386* | ++ |
| *Citrobacter koseri* LMGT 4063 | - |
| *Pseudomonas fluorescence* P1 LMGT 3020 | - |
| *P. aeruginosa* LMGT 3294 | - |
| *Escherichia coli* DH5α LMGT 3592 | - |
|  |  |

"-" no inhibition zone, "+" clear inhibition zone in diameter less or equal to 10 mm, "++" clear inhibition zone in diameter larger than 10 mm.
